# Risk perception and psychological state of healthcare workers in referral hospitals during the early phase of the COVID-19 pandemic, Uganda

**DOI:** 10.1186/s40359-021-00706-3

**Published:** 2021-12-17

**Authors:** Richard Migisha, Alex Riolexus Ario, Benon Kwesiga, Lilian Bulage, Daniel Kadobera, Steven N. Kabwama, Elizabeth Katana, Alex Ndyabakira, Ignatius Wadunde, Aggrey Byaruhanga, Geofrey Amanya, Julie R. Harris, Arthur G. Fitzmaurice

**Affiliations:** 1 Uganda Public Health Fellowship Program, Kampala, Uganda; 2 Ministry of Health, Kampala, Uganda; 3 COVID-19 Response International Task Force, US Centers for Disease Control and Prevention, Kampala, Uganda

**Keywords:** COVID-19, Risk perception, Psychological distress, Healthcare workers, Uganda

## Abstract

**Background:**

Safeguarding the psychological well-being of healthcare workers (HCWs) is crucial to ensuring sustainability and quality of healthcare services. During the COVID-19 pandemic, HCWs may be subject to excessive mental stress. We assessed the risk perception and immediate psychological state of HCWs early in the pandemic in referral hospitals involved in the management of COVID-19 patients in Uganda.

**Methods:**

We conducted a cross-sectional survey in five referral hospitals from April 20–May 22, 2020. During this time, we distributed paper-based, self-administered questionnaires to all consenting HCWs on day shifts. The questionnaire included questions on socio-demographics, occupational behaviors, potential perceived risks, and psychological distress. We assessed risk perception towards COVID-19 using 27 concern statements with a four-point Likert scale. We defined psychological distress as a total score > 12 from the 12-item Goldberg’s General Health Questionnaire (GHQ-12). We used modified Poisson regression to identify factors associated with psychological distress.

**Results:**

Among 335 HCWs who received questionnaires, 328 (98%) responded. Respondents’ mean age was 36 (range 18–59) years; 172 (52%) were male. The median duration of professional experience was eight (range 1–35) years; 208 (63%) worked more than 40 h per week; 116 (35%) were nurses, 52 (14%) doctors, 30 (9%) clinical officers, and 86 (26%) support staff. One hundred and forty-four (44%) had a GHQ-12 score > 12. The most common concerns reported included fear of infection at the workplace (81%), stigma from colleagues (79%), lack of workplace support (63%), and inadequate availability of personal protective equipment (PPE) (56%). In multivariable analysis, moderate (adjusted prevalence ratio, [aPR] = 2.2, 95% confidence interval [CI] 1.2–4.0) and high (aPR = 3.8, 95% CI 2.0–7.0) risk perception towards COVID-19 (compared with low-risk perception) were associated with psychological distress.

**Conclusions:**

Forty-four percent of HCWs surveyed in hospitals treating COVID-19 patients during the early COVID-19 epidemic in Uganda reported psychological distress related to fear of infection, stigma, and inadequate PPE. Higher perceived personal risk towards COVID-19 was associated with increased psychological distress. To optimize patient care during the pandemic and future outbreaks, workplace management may consider identifying and addressing HCW concerns, ensuring sufficient PPE and training, and reducing infection-associated stigma.

## Introduction

During major outbreaks, healthcare workers (HCWs) may experience high levels of psychological stress [1, 2]. Depression, stress, anxiety, stigma arising from contracting infection, and concerns about infection including fear of infecting colleagues, friends, and family have all been reported previously among HCWs during the 2003 SARS outbreak [3–6] and during the Ebola Virus Disease outbreak in West Africa [7]. Such psychological distress can interfere with the effective implementation and sustainability of health services [8].

During the COVID-19 pandemic, an increased risk for short- and long-term mental health problems, including psychological distress has been reported among healthcare professionals involved in the management of patients with COVID-19 [9]. Recent evidence from various studies which have assessed the psychological impact of COVID-19 on HCWs, including systematic reviews, shows that psychological problems, such as psychological distress, insomnia, depression, and anxiety, have been very frequent among HCWs across the globe; prevalence estimates of these psychological problems that range from 39 to 71% have been reported [1, 9–14]. Nonetheless, most public health responses tend to focus primarily on the biological or physical effects of epidemics, ignoring the psychological effects of most disease outbreaks, despite the fact that the consequences are detrimental [15]. In low-income countries such as Uganda where there are limited human resources for health (approximately one skilled HCW for every 1000 persons as of 2019) [16], protecting the mental health of HCWs during outbreaks is especially important to ensure sustainability of healthcare services.

After confirming the first COVID-19 case in Uganda on March 21, 2020 [17], the number of confirmed COVID-19 cases increased to 212 with no deaths as of May 24, 2020 [18]. Although no HCWs in Uganda had been diagnosed with the disease at that time [18], there were widespread reports globally about HCWs who had contracted the disease and died [19–22]. HCWs were reportedly at higher risk both for disease and death [23, 24], and heightened tension and fear were anticipated among HCWs in Uganda. To understand more about potential psychological distress among HCWs in Uganda and recommend appropriate interventions, we assessed risk perception and immediate psychological state among HCWs with regard to the COVID-19 outbreak.

## Methods

### Study design and population

We conducted a cross-sectional survey from April 20–May 22, 2020, in Central (Mulago National Referral Hospital, Entebbe Regional Referral Hospital), Eastern (Jinja Regional Referral Hospital), Western (Kabale Regional Referral Hospital), and Northern (Arua Regional Referral Hospital) regions of Uganda. At the time of this study, these hospitals were the only hospitals managing active COVID-19 case-patients. By the time the study began, the hospitals had managed 212 cases [18].

We designed a self-administered, structured questionnaire based on previous studies in outbreaks of respiratory infectious diseases, including COVID-19 in China [1, 25–27]. We chose a convenience sample of HCWs (doctors, clinical officers, nurses, midwives, radiographers, cleaners, drivers, administrators, laboratory personnel, and support staff) present on day-shift duties who consented to participate in the survey. The number of questionnaires distributed was based on the number of HCWs on duty in the respective hospitals (total of 335). Recruitment took one day in each referral hospital.

The principal investigator explained the study purpose and procedures to the HCWs in the respective departments and obtained written informed consent from all the participants prior to participation in the study. The participants indicated their consent by checking an appropriate box for consent before filling the questionnaire. The number of questionnaires distributed was based on the number of HCWs on duty, as determined by the respective heads of departments, in the respective hospitals. The questionnaires were returned by the heads of departments after 24 h. HCWs were categorized as ‘direct contact group’ if their jobs involved direct contact with patients and ‘indirect contact group’ if they were in contact with patient-related items (e.g., biological specimens, equipment), as defined previously [27].

### Study variables and data collection instruments

We captured data on HCWs’ socio-demographic and occupational characteristics, concerns and attitudes regarding COVID-19, and their immediate psychological status. Data collected included age, sex, professional cadre, level of education, years of professional experience, number of hours worked per week, number of children, persons with whom the HCW resided, and whether the HCW had ever provided care to a suspected or confirmed COVID-19 patient.

We assessed risk perception towards COVID-19 using 27 concern statements related to fear of contracting COVID-19, fear of spreading COVID-19, workplace-related conditions, and stigma. Each concern statement had four response options: ‘strongly agree,’ ‘agree,’ ‘disagree,’ or ‘strongly disagree’. We applied a scoring system using a four-point Likert scale from zero points (‘strongly disagree’) to three points (‘strongly agree’). Concern statements were negatively-worded (e.g., “there is no adequate personal protective equipment (PPE) at my workplace”), so that a higher score signified a higher degree of risk perception.

We used the 12-item General Health Questionnaire (GHQ-12) developed by Goldberg to assess the psychological state of HCWs [28]. The tool is multi-dimensional and has questions that assess social dysfunction, anxiety, and depression. The GHQ-12 has been widely used in assessing psychological state in outbreaks of infectious respiratory diseases (e.g., SARS and COVID-19) and found to have high reliability and validity [1, 5, 29–31]. The instrument includes 12 items (six negatively-worded and six positively-worded). The scoring method (from 0 to 36) is described elsewhere [32]. Briefly, we adopted the four-point Likert scale, with each item score ranging from ‘0’ to ‘3’. For negatively-worded items, ‘0’ indicated ‘Not at all’, ‘1’ indicated ‘No more than usual’, ‘2’ indicated ‘Rather more than usual’ and ‘3’ indicated ‘Much more than usual’. Positively-worded items were scored as follows: ‘0’ indicated ‘More so than usual’, ‘1’ indicated ‘Same as usual’, ‘2’ indicated ‘Less so than usual’, and ‘3’ indicated ‘Much less than usual’. All items were added to obtain the total score, ranging from 0 to 36 (with a higher score signifying worse mental health status). We classified respondents with scores greater than the cut-off point of 12 as having psychological distress, as previously described [33].

### Data management and statistical analysis

We entered data into EpiData 3.1 (EpiData, Odense, Denmark) and exported it to STATA version 13 (Statacorp, College Station, Texas) for analysis. Categorical data were summarized by frequencies and percentages; continuous normally-distributed data (risk perception score and GHQ-12 score) were presented as means with standard deviations (SDs), and continuous non-normally-distributed data (hours worked, number of children) as medians with interquartile ranges.

We dichotomized responses to concern statements into non-concern (strongly disagree and disagree) and concern (strongly agree and agree). Respondents were categorized into three groups: low risk perception (at or below the first quartile of concern scores); moderate risk perception (in the second quartile); and high-risk perception (third and fourth quartiles), as used previously [27]. The prevalence of psychological distress was determined as the percentage of respondents with GHQ-12 score greater than 12.

Finally, we performed univariable and multivariable analyses with psychological distress as a binary outcome to identify factors associated with psychological distress among HCWs. We considered risk perception among HCWs as our main exposure variable of interest and adjusted for other variables, including duration of professional experience, contact with confirmed COVID-19 case, and sex as potential confounders.

We reported prevalence ratios (PRs) with corresponding 95% confidence intervals (CIs) as measures of association between psychological distress and associated factors. We obtained PRs via modified Poisson regression, using a generalized linear model with Poisson as family and a log link without an offset but including robust standard errors. We did not use odds ratios as the measures of association because they could potentially overestimate the effect given the high prevalence of our outcome variable.

## Results

### Socio-demographics

Among 335 HCWs who received questionnaires, 328 (98%) completed and returned them. The remaining seven HCWs returned the questionnaires unfilled; none of the HCWs declined. Respondents’ mean age was 36 (SD ± 9.9) years and ranged from 18 to 59 years. Approximately half were male and half female. More than one-third had fewer than five years of work experience (median eight years, range 1–35 years), and three-quarters worked in direct contact with patients. Most worked more than 40 h per week (63%) (median 50, range 24–104) and had a child or children (69%). Approximately half reported ever providing direct care to suspected (57%) or confirmed (46%) COVID-19 cases at the time of study early in the pandemic (Table 1).

**Table 1:** Characteristics of respondents during a study to assess the psychological impact of COVID-19 on healthcare workers early in the COVID-19 epidemic, Uganda (N = 328)

Characteristic	Total (N = 328)	
	Number	Percent
*Health facility location*		
Jinja	88	27
Entebbe	81	25
Arua	72	22
Kabale	57	17
Mulago	30	9.1
*Age in years*		
18–35	174	53
≥ 36	154	47
*Sex*		
Male	172	52
Female	156	48
*Cadre of healthcare workers*		
Nurse	116	35
Support staff*	86	26
Doctor	52	14
Clinical officer	30	9.0
Midwife	21	6.4
Laboratory personnel	17	5.2
Pharmacist	5	1.5
Radiographer	1	0.3
*Category by patient contact*		
Direct contact group	242	74
Indirect contact group	86	26
*Had provided direct care to suspected COVID-19 case*	186	57
*Had provided direct care to confirmed COVID-19 cas*e	151	46
*Years of experience*		
< 5	124	38
5–10	89	27
> 10	115	35
*Hours worked per week*		
≤ 40	120	37
> 40	208	63
*Highest level of qualification*		
None	11	3.4
Certificate	77	23
Diploma	101	31
Degree	110	34
Masters	15	4.6
Others (Post-Masters’ and PhD Fellowships)	14	4.3
*Marital status*		
Single	120	36
Married/living with a partner	199	61
Separated/divorced	9	2.7
Has child or children	225	69
*With whom the healthcare worker stays at home*		
Family	212	65
Alone	90	27
Others	26	7.9

*Support staff included cleaners, ambulance drivers, and administrators

### Level of perceived risk towards COVID-19

**Fig. 1:**
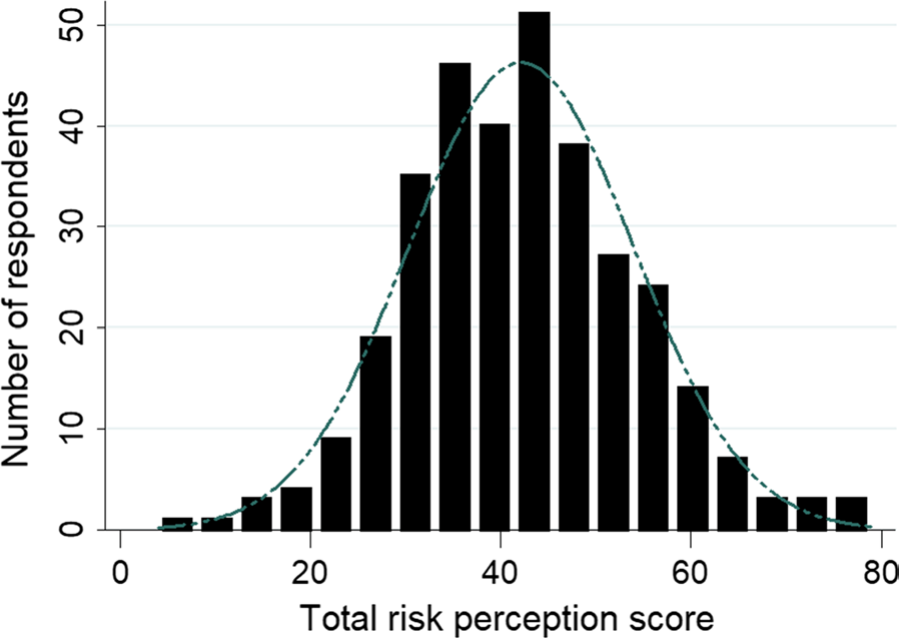
Distribution of total risk perception score derived from concern statements in tertiary referral hospitals during the early phase of the COVID-19 epidemic, Uganda, April–May 2020 (N = 328). Note: Higher risk perception scores signify higher degree of concern among the healthcare workers

The possible range of total concern scores reported by our respondents was 0–81 points. The mean risk perception score derived from the concern statements was 42 (SD ± 12), ranged from 4 to 79 points, and was normally-distributed (Fig. 1). For the direct contact group (n = 242), the mean score was 42 (SD ± 12), while for the indirect contact group (n = 86) the mean score was 43 (SD ± 11).

The greatest concerns of the group were about exposure to or illness with COVID-19, reflected in feeling endangered if a colleague contracted COVID-19 (89%), feeling at risk of contracting COVID-19 at the workplace (81%), feeling ashamed disclosing to colleagues if they contracted COVID-19 (79%), and feeling that they should observe social distancing more than non-HCWs (75%) (Table 2). Many also reported increased workload (59%) and inadequate staffing (58%) as problems. Respondents were least concerned about stigma from their families and COVID-19 standard operating procedures at their workplaces. Approximately one quarter of the respondents reported that they would feel ashamed disclosing to family if they contracted COVID-19 (24%), felt they should change their job due to COVID-19 risk (23%), did not feel safe with standard infection prevention and control (IPC) measures (25%), and were worried about the workplace not having a clear outbreak response plan (26%) (Table 2).

**Table 2:** Concerns of healthcare workers with regard to COVID-19 outbreak during the early phase of the epidemic, Uganda, April–May 2020 (N = 328)

Concern statement	Responses to concern statements (N = 328)			
	Number		Percent	
	Low concern	High concern	Low concern	High concern
*Fear of contracting COVID-19 at workplace*				
I would feel endangered if a colleague contracted COVID-19	35	293	11	89
I am at risk of contracting COVID-19	63	265	19	81
I feel anxious at work	96	233	21	71
I am unsafe at work	95	232	29	71
I will eventually get COVID-19	191	137	58	42
Being absent will reduce my chances of contracting COVID-19	201	127	61	39
I feel helpless about contracting COVID-19	213	109	67	33
I feel I should avoid going to work to avoid contracting COVID-19	230	98	70	30
I do not feel safe even when I use standard IPC measures	245	83	75	25
I feel I should change job in future due to COVID-19 risk	253	75	77	23
*Perceived workplace risks and conditions*				
My workplace would not support me if I contracted COVID-19	120	208	37	63
COVID-19 outbreak has increased my workload	134	194	41	59
Workload is not matched with staffing needs	137	191	42	58
There is no adequate PPE	143	185	44	56
I have not received adequate training on IPC	189	139	58	42
I feel overwhelmed by new COVID-19 regulations	193	135	49	41
I am not confident about IPC measures	216	112	66	34
There is no clear outbreak response plan	243	85	74	26
*Fear of spreading COVID-19*				
I should social distance more than non-HCWs	82	246	25	75
I will likely transmit COVID-19 to family members	132	196	40	60
*Stigma against self (internal) and others (external)*				
I would feel ashamed disclosing to colleagues if I contracted COVID-19	69	259	21	79
Family will not look after me if I contract COVID-19	153	175	47	53
I feel forced to care for COVID-19 patients	173	155	53	47
I would feel ashamed disclosing to my family if I contracted COVID-19	249	79	76	24

*IPC* infection control and prevention, *HCW* healthcare worker, *COVID-19* coronavirus disease, *PPE* personal protective equipment

### Level of psychological distress

The mean GHQ-12 distress score of the HCWs was 12 (SD ± 7.2); 144 had a GHQ-12 score > 12, yielding a prevalence of psychological distress of 44% (95% CI 38–49%). The most commonly-reported indicators from the GHQ-12 questionnaire with a score > 1 were not enjoying day-to-day activities (54%), constantly feeling under stress (50%), not feeling reasonably happy (43%), and feeling unhappy and depressed (40%). Approximately three in ten (29%) reported losing sleep, and two in ten (22%) reported feeling worthless (Table 3).

**Table 3:** Frequency of GHQ-12 items among healthcare workers in tertiary referral hospitals during the early phase of the epidemic, Uganda, April–May 2020 (N = 328)

Items^†^ from GHQ-12 questionnaire^§^	Percentage of score responses (N = 328)				
	0	1	2	3	2 or 3*
Not enjoying daily activities	21	25	25	29	54
Feeling constantly under stress	37	14	22	28	50
Not feeling reasonably happy	20	37	21	22	43
Feeling unhappy and depressed	44	16	17	23	40
Cannot overcome difficulties	30	32	20	18	38
Cannot concentrate on tasks	39	30	20	11	31
Losing sleep worrying about COVID-19	55	16	8.8	20	29
Cannot face up to problems	41	34	16	9.8	26
Lost confidence	61	15	12	13	25
Feeling worthless	70	7.9	10	12	22
Not capable of making decisions	47	34	15	4	19
Not feeling useful in society	73	19	3.4	4.3	8

*A higher score signifies psychologically-distressed state; GHQ: General health questionnaire

^†^All items were asked about for the period of the past one month

^§^GHQ-12 items as proposed by Goldberg [19]

### Factors associated with psychological distress

Compared to HCWs with a low risk perception score towards COVID-19, the prevalence of psychological distress was significantly higher among those with moderate (aPR = 2.2; 95% CI 1.2–4.0) and high (aPR = 3.8; 95% CI 2.0–7.0) risk perception towards COVID-19 (Table 4).

**Table 4:** Factors associated with psychological distress among healthcare workers in tertiary referral hospitals during the early phase of the COVID-19 epidemic, Uganda, April–May 2020 (N = 328)

Characteristic	Psychological distress		Univariable analysis		Multivariable analysis**	
	Distressed (n = 144), n (%)	No distress (n = 184), n (%)	PR (95% CI)	*P* value	Adjusted PR (95% CI)	*P* value
*Level of concern/risk perception*						
Low	13 (9.0)	59 (32)	Ref		Ref	
Moderate	72 (50)	103 (56)	2.3 (1.3–4.1)	0.006	2.2 (1.2–4.0)	0.010
High	59 (41)	22 (12)	4.0 (2.2–7.4)	< 0.001	3.8 (2.0–7.0)	< 0.001
*Age category*						
≤ 35 years	73 (51)	101 (55)	Ref			
> 35 years	71 (49)	83 (45)	1.1 (0.79–1.5)	0.571		
*Sex*						
Male	67 (47)	105 (57)	Ref		Ref	
Female	77 (53)	79 (43)	1.3 (0.98–1.8)	0.156	1.2 (0.84–1.7)	0.322
*Category of HCW by patient contact*						
Direct contact	110 (76)	132 (72)	Ref			
Indirect contact	34 (24)	52 (28)	0.87 (0.60–1.3)	0.477		
*Years of experience*						
< 5 years	46 (32)	78 (42)	Ref		Ref	
5–10 years	43 (30)	46 (25)	1.3 (0.86–2.0)	0.213	1.3 (0.84–1.9)	0.253
> 10 years	55 (38)	60 (33)	1.3 (0.87–1.9)	0.204	1.1 (0.79–1.6)	0.778
*Hours worked per week*						
≤ 40 h	58 (40)	62 (34)	Ref			
> 40 h	86 (60)	122 (66)	0.86 (0.61–1.2)	0.358		
*Number of children*						
None	45 (31)	58 (32)	Ref			
One or more	99 (69)	126 (69)	1.0 (0.71–1.4)	0.969		
Provided direct care for suspected COVID-19 case*						
No	71 (49)	71 (39)	Ref			
Yes	73 (51)	113 (61)	0.78 (0.57–1.1)	0.146		
*Provided direct care for confirmed COVID-19 case*						
No	91 (63)	86 (47)	Ref		Ref	
Yes	53 (37)	98 (53)	0.68 (0.49–0.96)	0.027	0.87 (0.61–1.2)	0.430

*Ref* reference category, *CI* confidence interval, *PR* prevalence ratio, *HCW* healthcare worker

*Excluded from multivariable model due to collinearity with provision of direct care to confirmed case

**Adjusted for sex, years of professional experience, and providing direct care to a confirmed COVID-19 case

In univariable analyses, none of the socio-demographic or occupational factors, including age category, sex, HCW category by patient contact (direct/indirect), duration in service, working hours, having children, or experience providing direct care to a suspected COVID-19 case were significantly associated with psychological distress. Experience providing direct care to a confirmed COVID-19 case did not have a significant effect on psychological distress prevalence when controlling for other potential factors, including risk perception score.

## Discussion

We assessed risk perception and psychological state of HCWs based in referral hospitals designated to manage COVID-19 patients in the early phase of the COVID-19 epidemic in Uganda. We found psychological distress in 44% of the HCWs surveyed in the first two months (April–May 2020) of the epidemic. The level of risk perception towards COVID-19 was directly and independently associated with psychological distress among HCWs.

Reports of psychological distress among HCWs during the COVID-19 pandemic have varied. The prevalence of distress reported in the current study is comparable to the prevalence of psychological distress of 39% reported among HCWs in China in the early phase of the pandemic [1], but much lower than the 72% prevalence reported among HCWs in high-risk situations in China when the total confirmed cases had already surpassed 10,000 in the country [14]. The lower prevalence of psychological distress in our study compared to China may relate to the fact that none of the HCWs in Uganda had been diagnosed with COVID-19 at the time of the survey [18]. Furthermore, the incidence of COVID-19 was substantially lower in Uganda than in China, which may have accounted for the difference [17]. Notably, we found higher levels of psychological distress among healthcare workers during the COVID-19 pandemic than were reported in Hong Kong (7%) and Canada (29%) during the earlier SARS outbreak [6, 29]. It is possible that the transmissibility of SARS-CoV-2 in the absence of symptoms, which prevents easy identification of infected persons, may have increased the level of concern among HCWs.

We found a strong association between risk perception towards COVID-19 and psychological distress among HCWs in Uganda. This is both expected and consistent with studies of HCWs’ distress during outbreaks of other respiratory infectious diseases [1, 25, 27, 34–36]. Others have noted that HCWs’ psychological distress can derive from managing the dynamics of challenges to personal safety, fear for others or oneself becoming infected, and altruism and professional responsibility [15, 37]. Concerns about the safety of HCWs or their families and friends, changes in workplace dynamics, and being isolated can be major sources of distress [38].

Our findings point towards potential interventions to address the concerns of HCWs in Uganda and improve their psychological well-being. About half of respondents reported inadequate PPE availability, while most felt safe when using IPC measures. The fear of contracting infection among HCWs might have been heightened in facilities with inadequate PPE stock and might have increased as PPE was used and shortages were anticipated. To protect the physical and mental health of HCWs, the Ministry of Health (MoH) and facility management may consider maintaining adequate PPE supply and facilitating routine IPC trainings.

More than half of the HCWs also reported increased workloads and inadequate staffing. Employers may consider setting shorter working hours, rotating shifts for HCWs working in high-risk zones, and/or encouraging regular rest periods, when possible, to improve the morale of HCWs during the pandemic [15]. In circumstances where shortening of working hours in epidemics is not a feasible option, employers could consider other options including providing incentive pay for extra hours worked and offering complimentary transportation and food for HCWs on duty. Such practices are particularly important in epidemics that extend several months or even years when HCWs will feel the long-term effects of overworking [15].

Others have also reported that inclusive leadership can alleviate the psychological distress of HCWs [39]. This can mean providing HCWs with opportunities to share their concerns so they can be directly addressed. Most respondents expressed perceived stigma if they had to disclose contracting COVID-19 to colleagues. Supervisors and employers should make deliberate efforts to render more psychosocial support to HCWs who may contract COVID-19 and to regard such infections as work-related injuries. In this context, in which HCWs reported concerns about not being supported by their workplace if they contracted COVID-19, the MoH or facility management may consider providing HCWs with healthcare and compensation and assure them they would not lose their jobs if they get infected during the epidemic. Additionally, peer support systems for HCWs could be established and HCWs encouraged to utilize them for psychological distress to be identified and addressed in a timely manner without HCWs perceiving stigma or discrimination. These practices could reduce the psychological impact in a healthcare work environment during epidemics [15]. Our findings that a meaningful proportion of the HCWs had insomnia and felt worthless and depressed suggests that the HCWs could experience long-term mental health problems, as was observed in similar previous outbreaks including the 2003 SARS outbreak [40]. We recommend follow-up studies to assess mental health outcomes related to COVID-19 among the HCWs, in order to inform timely interventions. In the meantime, the MoH may consider setting up psychological support networks nationwide including internet-, or telephone-based counselling/treatment services targeting HCWs as part of an epidemic response.

Our findings are subject to three main limitations. First, we relied on self-report of psychological status and risk perception, so these findings may be prone to response bias including social desirability bias, although this was minimized by using self-administered questionnaires. This may have led to underestimation of the prevalence of psychological distress in the study population, and potentially biased our associations towards null. Second, we included only the day-shift employees available at our visits. These represented approximately one-third of HCWs at the facilities and may not be representative of all employees at the hospitals. Third, our study lacked a comparison group of HCWs in hospitals that were not treating COVID-19 patients at the time; we were, therefore, unable to compare the level of psychological distress between the two groups. Despite these limitations, our survey provided useful information to the MoH on the psychological state of HCWs and highlighted their key concerns in the first two months of the COVID-19 outbreak in Uganda; this informed designing of evidence-based measures to improve HCWs’ psychological well-being during the pandemic, especially by improving supplies of PPE and conducting IPC trainings to HCWs.

## Conclusion

About half of the HCWs surveyed in the early phase of the COVID-19 epidemic in Uganda reported psychological distress even before any HCWs in Uganda had contracted COVID-19. The perceived personal risk was associated with psychological distress, so reducing perceived risks might enhance HCWs’ physical and psychological well-being. This work reveals several HCWs’ concerns that might be addressed to improve the psychological health of HCWs during this ongoing pandemic and in future epidemics. This might be accomplished by ensuring sufficient PPE and access to IPC training, improving morale, addressing stigma in the workplace and in the community, and rendering more psychosocial support by employers and supervisors. Follow-up studies in different phases during and after the COVID-19 pandemic might further reveal impacts of COVID-19 on HCWs’ stress and the effectiveness of practices aimed at strengthening their mental health and occupational safety; such psychological distress may interfere with the effective implementation and sustainability of health services. Qualitative interviews might particularly help elucidate the nature and extent of the psychological impact of the COVID-19 pandemic on HCWs.

## Data Availability

The datasets upon which our findings are based belong to the Uganda Public Health Fellowship Program. For confidentiality reasons, the datasets are not publicly available. However, the data sets can be availed upon reasonable request from the corresponding author and with permission from the Uganda Public Health Fellowship Program.

## Abbreviations

CI: Confidence interval
COVID-19: Coronavirus Disease
GHQ: General health questionnaire
HCW: Healthcare worker
IQR: Inter-quartile Range
MoH: Ministry of Health
PPE: Personal protective equipment
PR: Prevalence ratio
SARS-CoV-2: Severe Acute Respiratory Syndrome Coronavirus 2
SD: Standard Deviation
